# Comparative Discrimination of Boey, PULP, and PPUMSs for Textbook Outcome Failure, Failure-to-Rescue, and 30-Day Mortality Following Perforated Peptic Ulcer Surgery

**DOI:** 10.3390/medicina62101847

**Published:** 2026-09-24

**Authors:** Burak Aydoğan, Uğur Topal, Serdar Gümüş, Yunus Kaycı, Veysel Demiroğlu, Mevlüt Harun Ağca, İshak Aydın, Ahmet Gökhan Sarıtaş, Orçun Yalav, Cem Kaan Parsak

**Affiliations:** 1 Department of General Surgery, Islahiye State Hospital, 27800 Gaziantep, Turkey; drburakaydogan@gmail.com; 2 Department of Surgical Oncology, Faculty of Medicine, Çukurova University, 01330 Adana, Turkey; 3 Department of Gastroenterological Surgery, Adana City Training and Research Hospital, University of Health Sciences, 01370 Adana, Turkey; 4 Department of General Surgery, Faculty of Medicine, Çukurova University, 01330 Adana, Turkey

**Keywords:** perforated peptic ulcer, Textbook Outcome, Failure-to-Rescue, risk score, PULP score, emergency surgery

## Abstract

*Background and Objectives*: Early Textbook Outcome (Early TO), defined via the Naumann/BEACON Delphi process for non-traumatic emergency laparotomy, and Failure-to-Rescue (FTR) provide postoperative outcome frameworks beyond overall mortality. This study compared the discriminative performance of the mortality-oriented Boey, Peptic Ulcer Perforation (PULP), and Perforated Peptic Ulcer Mortality Score (PPUMS) scores for Early TO failure, FTR, and 30-day mortality in perforated peptic ulcer surgery. *Materials and Methods*: This retrospective, single-center cohort study included 128 consecutive adults undergoing emergency surgery for gastroduodenal peptic ulcer perforation (January 2015–February 2026). Boey, PULP, and PPUMSs were calculated from preoperative data. The primary outcome was Early TO failure (Naumann/BEACON criteria); secondary outcomes were FTR and 30-day mortality. FTR was assessed among patients with a documented Clavien–Dindo grade III/IV complication. Discriminative performance was quantified as the area under the receiver operating characteristic curve (AUC); correlated AUCs were compared using paired DeLong tests, with Holm adjustment for multiple comparisons within each outcome. *Results*: Early TO failure occurred in 47 patients (36.7%) and completely overlapped with Clavien–Dindo grade ≥ III postoperative morbidity; no additional patient was classified as Early TO failure solely by another Early TO component. The exploratory FTR analysis included 47 patients with a major postoperative complication, of whom 30 (63.8%) experienced FTR; 30-day mortality in the full cohort was 23.4%. PULP yielded the highest AUCs and Boey the lowest across outcomes: for Early TO failure, AUCs were 0.749 (95% CI, 0.660–0.838) for PULP, 0.703 (0.611–0.795) for PPUMS, and 0.587 (0.487–0.687) for Boey; corresponding AUCs were 0.832, 0.778, and 0.532 for FTR, and 0.851, 0.790, and 0.588 for 30-day mortality. After Holm adjustment, PULP outperformed Boey for all three outcomes, and PPUMS outperformed Boey for FTR and 30-day mortality; the PPUMS–Boey difference was not significant for Early TO failure (*p* = 0.057). PULP and PPUMS did not differ significantly for any outcome. *Conclusions*: PULP yielded numerically higher AUCs than PPUMS across all three analyses, although none of the PULP–PPUMS differences reached statistical significance; equivalence between the two scores cannot be inferred. In this cohort, Early TO failure completely overlapped with Clavien–Dindo grade ≥ III postoperative morbidity; therefore, its discriminative performance primarily reflected major postoperative morbidity rather than an independent composite outcome. FTR-specific findings should be considered exploratory and should not be interpreted as measures of institutional rescue capability. Prospective multicenter validation incorporating formal calibration and clinical utility assessment is required before direct clinical implementation.

## 1. Introduction

Perforated peptic ulcer (PPU) remains associated with high postoperative morbidity and mortality despite advances in contemporary emergency surgical management [1,2]. The Boey and Peptic Ulcer Perforation (PULP) scores used in PPU surgery were developed and evaluated primarily for mortality prediction [3,4,5]. More recently, the Perforated Peptic Ulcer Mortality Score (PPUMS) was developed to predict the risk of in-hospital mortality [6]. Although survival is a fundamental outcome, it may not adequately reflect the quality of postoperative recovery when considered alone. Standardized outcomes that capture postoperative results beyond mortality may provide a more comprehensive assessment.

The Naumann/BEACON international Delphi process defined Early Textbook Outcome (Early TO) in patients undergoing non-traumatic emergency laparotomy as discharge from hospital without Clavien–Dindo grade III or higher (CD grade ≥ III) morbidity, intra-abdominal sepsis, organ failure, unplanned reoperation, or death [7]. Failure-to-Rescue (FTR) was originally defined as death following a postoperative complication and was subsequently used in surgical quality research to evaluate survival after major complications [8,9]. This conceptual framework provides a basis for evaluating mortality-oriented risk scores in relation to postoperative recovery and mortality following major postoperative complications.

The Boey, PULP, and PPUMSs used in PPU surgery partially overlap at the component level but represent different clinical risk constructs [3,6,10]. These scores are based on criteria that assign different weights to components such as comorbidity, the burden of peritonitis, and physiological derangement in mortality prediction [6,10,11]. These structural differences raise the question of how well the scores discriminate across postoperative outcomes beyond their original mortality endpoints.

To our knowledge, the comparative discriminative performance of these scores for Early TO failure and FTR has not yet been established in the existing literature. Although morbidity prediction has previously been investigated in PPU surgery, the outcomes evaluated have ranged from postoperative morbidity and 30-day morbidity to a high cumulative complication burden [12,13,14]. These studies did not evaluate postoperative recovery and mortality following major complications within a common comparative framework [12,13,14]. A recent systematic review and meta-analysis compared the accuracy of the Boey and PULP scores for conventional mortality and morbidity prediction but did not evaluate FTR or Naumann-defined Early TO failure [15].

This study aimed to evaluate the comparative discriminative performance of the Boey, PULP, and PPUMSs for Early TO failure, FTR, and 30-day mortality.

## 2. Materials and Methods

### 2.1. Study Design and Patient Selection

This retrospective, single-center cohort study included consecutive adult patients who underwent emergency surgery for gastroduodenal peptic ulcer perforation at Çukurova University Faculty of Medicine Hospital between January 2015 and February 2026. Eligibility criteria were age ≥ 18 years, intraoperative confirmation of gastric or duodenal peptic ulcer perforation, and emergency operative treatment. Patients with incomplete retrospective records that precluded reliable calculation of the Boey, PULP, and PPUMSs were excluded. The pattern of missing data among excluded patients was summarized at the variable level. Available characteristics of excluded patients were compared with those of included patients when data were available, and no imputation was performed because score calculation was restricted to directly observed score components (Supplementary Table S1). No a priori sample-size calculation was performed; the final sample was determined by the number of eligible patients with complete data required for score calculation during the predefined study period. The patient selection process is shown in Figure 1. The study was reported in accordance with the STROBE recommendations and the applicable TRIPOD items for studies evaluating prediction model performance.

### 2.2. Data Collection and Clinical Variables

Demographic characteristics, body mass index, comorbidities, American Society of Anesthesiologists (ASA) physical status class, medication use, time of symptom onset, times of hospital admission and surgery, preoperative vital signs, and laboratory values were retrospectively collected from electronic hospital records, operative records, and archived patient files. ASA physical status was obtained directly from the contemporaneous preoperative anesthesia assessment and was not retrospectively reconstructed. Systolic blood pressure and heart rate recorded at hospital admission and the first available preoperative laboratory results were used for score calculation. The first available preoperative laboratory values were used to standardize retrospective score reconstruction and minimize the influence of subsequent treatment-related changes. Symptom onset was defined as the patient-reported onset of symptoms consistent with the acute perforation episode, as documented in the emergency department or admission records, rather than a clinician-estimated time of perforation. When an exact onset time was unavailable, an approximate duration was used only if the documentation permitted unambiguous classification relative to the prespecified 24 h threshold; otherwise, the interval was considered unavailable and was not inferred. These intervals were used for the time-dependent components of the Boey and PULP scores. Data extraction was performed by one investigator (V.D.), and the extracted data and calculation of the Boey, PULP, and PPUMSs were independently reviewed and verified by a second investigator (B.A.). No discrepancies were identified.

Operative variables included the gastric or duodenal location of the perforation, perforation diameter, surgical approach, and procedure performed. Postoperative data included complications, requirement for intensive care, unplanned reoperation, length of hospital stay, discharge status, and death within 30 days after surgery. For patients discharged alive before postoperative day 30, vital status through postoperative day 30 was verified from subsequent hospital records. All such patients had a documented hospital encounter after postoperative day 30, confirming survival through the 30-day follow-up period. Accordingly, 30-day vital status was completely ascertained for all 128 patients included in the analytic cohort. Postoperative complications were graded according to the Clavien–Dindo (CD) classification, and the highest grade recorded during the index hospitalization was used [16].

### 2.3. Calculation of Risk Scores

The Boey score, Peptic Ulcer Perforation (PULP) score, and Perforated Peptic Ulcer Mortality Score (PPUMS) were retrospectively calculated using preoperatively available clinical data according to the original scoring systems and prespecified operational definitions [3,5,6]. The original score components, operational definitions applied in the present study, and corresponding data sources are summarized in Supplementary Table S2.

### 2.4. Boey Score

The Boey score comprises three components: major medical illness, preoperative shock, and prolonged perforation duration. One point was assigned for the presence of each component. The major medical illness component was operationalized for the available dataset based on the original categories of cardiorespiratory disease, renal failure, diabetes, and hepatic precoma. This component was considered present when at least one of the following conditions was documented: coronary artery disease or previous myocardial infarction, congestive heart failure, chronic obstructive pulmonary disease, pre-existing chronic kidney disease, or diabetes mellitus. Liver cirrhosis without hepatic precoma or encephalopathy was not included in this component.

Preoperative shock was defined as systolic blood pressure < 100 mmHg. Prolonged perforation duration was operationalized as an interval of >24 h from the onset of symptoms consistent with acute perforation to surgery. The total Boey score ranged from 0 to 3.

### 2.5. PULP Score

The PULP score was calculated according to the original scoring system. Three points were assigned for age > 65 years; 1 point for active malignancy or acquired immunodeficiency syndrome; 2 points for liver cirrhosis; 1 point for concomitant steroid use; 1 point for shock at admission; 1 point for an interval of >24 h from the onset of symptoms consistent with acute perforation to hospital admission; and 2 points for a serum creatinine level > 130 µmol/L, approximately 1.47 mg/dL. ASA classes I, II, III, IV, and V were assigned 0, 1, 3, 5, and 7 points, respectively. Shock was defined as systolic blood pressure < 100 mmHg with a concurrent heart rate > 100 beats/min. The score-specific definitions of preoperative shock were preserved from the original scoring systems. The total PULP score ranged from 0 to 18.

### 2.6. PPUMS

The PPUMS was calculated using age categories and five comorbidity components. Mutually exclusive age categories were defined as <45, 45–64, 65–80, and >80 years and assigned 0, 1, 2, and 3 points, respectively. Congestive heart failure, severe liver disease, chronic kidney disease, a history of malignancy, and obesity were each assigned 1 point.

Severe liver disease was defined as the presence of at least one of the following: intractable ascites, hepatic encephalopathy, or bleeding from esophageal varices. Renal disease was defined as pre-existing chronic kidney disease. An isolated elevation in serum creatinine at admission was not scored as renal disease. Because obesity was identified using diagnostic codes in the original PPUMS study, this component was operationalized in the present clinical dataset as a body mass index ≥ 30 kg/m^2^. The total PPUMS ranged from 0 to 8.

### 2.7. Study Outcomes

The primary outcome was failure to achieve Early TO, as defined by Naumann et al. for patients undergoing non-traumatic emergency laparotomy [7]. Early TO was defined as discharge without CD grade ≥ III postoperative morbidity, intra-abdominal sepsis, organ failure, unplanned reoperation, or death. For the present study, the death component was operationalized as all-cause mortality occurring within 30 days after surgery. The presence of any of these components was considered Early TO failure. Component events were not mutually exclusive.

The secondary outcomes were FTR and 30-day mortality. FTR was defined as death within 30 days after surgery among patients who developed a documented major postoperative complication [8,9]. Eligibility for the primary FTR analysis required a separately documented postoperative CD grade III or IV complication occurring before death or discharge. CD grade V was not used alone to establish eligibility for the FTR denominator because grade V denotes postoperative death itself rather than a preceding complication. For characterization of rescue according to complication severity, the highest separately documented CD grade III or IV complication occurring before death or discharge was used.

Thirty-day mortality was defined as all-cause death occurring within 30 days after surgery and was evaluated in the entire study cohort.

### 2.8. Statistical Analysis

Continuous variables were summarized as median and interquartile range, whereas categorical variables were presented as number and percentage. Continuous variables were compared between groups using the two-sided Mann–Whitney U test. Categorical variables were compared using the Pearson’s chi-square test, Fisher’s exact test, or the Fisher–Freeman–Halton exact test, as appropriate according to contingency-table dimensions and expected cell counts. For comparisons between included and excluded patients, formal hypothesis testing was restricted to characteristics available for all excluded patients, whereas characteristics with incomplete availability in the excluded group were summarized descriptively. The distributions of the Boey, PULP, and PPUMSs between outcome groups were evaluated using the two-sided Mann–Whitney U test.

The discriminative performance of the Boey, PULP, and PPUMSs for Early TO failure, FTR, and 30-day mortality was evaluated using receiver operating characteristic curves. Discrimination was expressed as the AUC with its 95% confidence interval. AUC values and their corresponding 95% confidence intervals were calculated using the DeLong method.

Pairwise differences between correlated AUCs obtained from the same patients were evaluated using the paired DeLong test. For each prespecified clinical outcome, the three pairwise comparisons among the Boey, PULP, and PPUMSs were treated as a separate family of hypotheses, and *p* values were adjusted within each outcome using the Holm method. For pairwise AUC comparisons, AUC differences with 95% confidence intervals and both unadjusted and Holm-adjusted *p* values were estimated. Statistical significance was determined according to the Holm-adjusted *p* values.

As a sensitivity analysis, FTR was additionally evaluated using a broader definition in which all patients with a final postoperative CD grade III, IV, or V were included in the FTR denominator, with CD grade V deaths classified as FTR events irrespective of whether a preceding grade III or IV complication had been separately documented. The FTR rate and the discriminative performance of the Boey, PULP, and PPUMSs under this broader definition were compared with those obtained using the primary FTR definition. Given the limited size of the FTR analysis population, all FTR-specific analyses were considered exploratory. The age-unadjusted Charlson Comorbidity Index (CCI) was included in the exploratory FTR subgroup analysis as a descriptive measure of baseline comorbidity burden and was not part of the primary comparison of the Boey, PULP, and PPUMSs.

Observed 30-day mortality proportions and 95% Wilson confidence intervals were calculated across predefined risk strata of the Boey, PULP, and PPUMSs. These proportions were compared descriptively with reference mortality estimates derived from the original score publications. For the PULP score, category-level reference values were calculated by weighting the published score-specific mortality proportions according to the score distribution within each category in the present cohort. These comparisons were descriptive; no formal calibration or clinical utility analyses were performed.

All tests were two-sided. A Holm-adjusted *p* value < 0.05 was considered statistically significant for pairwise AUC comparisons, whereas a two-sided *p* value < 0.05 was used for all other analyses. Statistical analyses were performed using Python 3.13.5 with SciPy 1.17.0 and statsmodels 0.14.6.

## 3. Results

### 3.1. Study Cohort and Outcome Frequencies

During the study period, 138 patients with perforated peptic ulcers were assessed for eligibility. Ten patients were excluded because incomplete retrospective records precluded reliable calculation of the Boey, PULP, and PPUMSs, leaving 128 patients in the final analytic cohort. Among the excluded patients, body mass index and steroid-use data were unavailable in all 10 patients, symptom-onset timing information in nine, and admission systolic blood pressure, heart rate, and serum creatinine in eight. Age, sex, and in-hospital mortality status were available for all 10 excluded patients. There were no statistically significant differences between included and excluded patients in age (67 [53–77] vs. 56.0 [54.0–62.5] years, *p* = 0.115) or sex (male, 68.8% vs. 50.0%, *p* = 0.294). No in-hospital deaths occurred among the excluded patients, compared with 30/128 (23.4%) among the included patients (*p* = 0.118). Thirty-day mortality could not be reliably ascertained for the excluded patients. The complete missing-data profile and comparison of available characteristics are presented in Supplementary Table S1. All patients were included in both Early TO and 30-day mortality analyses. Early TO was achieved in 81 patients (63.3%), whereas 47 patients (36.7%) experienced Early TO failure. At the patient level, Early TO failure completely overlapped with Clavien–Dindo grade ≥ III postoperative morbidity: all 47 patients with Early TO failure had a major postoperative complication, and no additional patient was classified as Early TO failure solely because of intra-abdominal sepsis, organ failure, unplanned reoperation, or death. These 47 patients constituted the denominator for the FTR analysis. Among these patients, 17 (36.2%) were successfully rescued, whereas 30 (63.8%) died within 30 days and were classified as FTR events. The overall 30-day mortality rate was 23.4% (30/128). All deaths occurred during the index hospitalization; therefore, in-hospital and 30-day mortality were identical (Figure 1).

### 3.2. Baseline and Perioperative Characteristics

Patients with Early TO failure were older than those who achieved Early TO (76 [63–82.5] vs. 61 [48–70] years, *p* < 0.0001), were less frequently male (55.3% vs. 76.5%, *p* = 0.013), and had higher ASA classes (3 [2,3] vs. 2 [2,3], *p* = 0.0004). Congestive heart failure was more frequent in the Early TO failure group (19.1% vs. 6.2%, *p* = 0.023). Patients with Early TO failure also had higher blood urea nitrogen and creatinine levels (both *p* < 0.0001) and lower serum albumin levels (*p* = 0.001). No significant between-group differences were observed in body mass index, diabetes mellitus, chronic obstructive pulmonary disease, chronic kidney disease, symptom duration, white blood cell count, perforation size or site, or operative procedure (Table 1). All 128 procedures were performed using an open approach; there were no laparoscopic procedures or conversions, and the operative approach remained unchanged throughout the study period.

### 3.3. Postoperative Outcomes

Postoperative outcomes and the individual components of Early TO failure are summarized in Table 2. Among the 47 patients with Early TO failure, all 47 (100.0%) had Clavien–Dindo grade ≥ III postoperative morbidity, 29 (61.7%) developed intra-abdominal sepsis, 32 (68.1%) developed organ failure, 10 (21.3%) underwent unplanned reoperation, and 30 (63.8%) died within 30 days. Components were not mutually exclusive. None of the non-morbidity components identified an additional patient with Early TO failure beyond those already classified as having CD grade ≥ III morbidity. Among these patients, 17 (36.2%) were rescued, whereas 30 (63.8%) experienced FTR. Rescue rates were 100.0% (13/13) for grade III, 26.7% (4/15) for grade IVa, and 0.0% (0/19) for grade IVb complications. Accordingly, all 30 deaths were preceded by a separately documented grade IV complication, comprising 11 grade IVa and 19 grade IVb events; thus, no deaths were excluded from the primary FTR analysis by the requirement for a qualifying grade III/IV complication. The 30-day mortality rate was 23.4%, and the median length of hospital stay was 9 [6–12] days.

In the sensitivity analysis using a broader FTR definition, in which all patients with a final postoperative CD grade III, IV, or V were included in the FTR denominator, the same 47 patients were identified, yielding an unchanged FTR rate of 63.8% (30/47). Because the analysis population and FTR classification were identical under the primary and broader definitions, the AUC estimates and pairwise score comparisons were also unchanged.

### 3.4. Risk-Score Distributions According to Outcome Status

PULP and PPUMSs were significantly higher in patients with Early TO failure than in those who achieved Early TO (both *p* < 0.0001). Among patients with major postoperative complications, both scores were also higher in patients who experienced FTR than in those who were successfully rescued (*p* = 0.0002 and *p* = 0.001, respectively). Similarly, PULP and PPUMSs were higher in patients who died within 30 days than in survivors (both *p* < 0.0001). Boey score distributions did not differ significantly according to Early TO status, FTR, or 30-day mortality (Table 3).

### 3.5. Discriminative Performance and Pairwise Score Comparisons

The discriminative performance of the Boey, PULP, and PPUMSs is presented in Table 4. PULP yielded the highest AUCs for Early TO failure, FTR, and 30-day mortality, with values of 0.749, 0.832, and 0.851, respectively. The corresponding AUCs for PPUMS were 0.703, 0.778, and 0.790, whereas Boey demonstrated lower discrimination across all three outcomes.

Pairwise comparisons of correlated AUCs showed that, for Early TO failure, the AUC difference was 0.162 (95% CI, 0.060–0.265; unadjusted *p* = 0.002; Holm-adjusted *p* = 0.006) for PULP versus Boey, 0.116 (95% CI, 0.012–0.221; unadjusted *p* = 0.028; Holm-adjusted *p* = 0.057) for PPUMS versus Boey, and 0.046 (95% CI, −0.014 to 0.106; unadjusted *p* = 0.135; Holm-adjusted *p* = 0.135) for PULP versus PPUMS. For FTR, the corresponding AUC differences were 0.300 (95% CI, 0.136–0.464; unadjusted *p* = 0.0003; Holm-adjusted *p* = 0.001), 0.246 (95% CI, 0.088–0.404; unadjusted *p* = 0.002; Holm-adjusted *p* = 0.004), and 0.054 (95% CI, −0.063 to 0.171; unadjusted *p* = 0.366; Holm-adjusted *p* = 0.366), respectively. For 30-day mortality, the corresponding differences were 0.263 (95% CI, 0.155–0.371; unadjusted *p* < 0.0001; Holm-adjusted *p* < 0.0001), 0.202 (95% CI, 0.093–0.312; unadjusted *p* = 0.0003; Holm-adjusted *p* = 0.0006), and 0.061 (95% CI, −0.007 to 0.128; unadjusted *p* = 0.079; Holm-adjusted *p* = 0.079), respectively. After Holm adjustment within each outcome, PULP demonstrated significantly greater discrimination than Boey for Early TO failure, FTR, and 30-day mortality. PPUMS also showed significantly greater discrimination than Boey for FTR and 30-day mortality; however, the difference for Early TO failure did not remain statistically significant after adjustment. No statistically significant differences were observed between PULP and PPUMS for any of the three outcomes. The receiver operating characteristic curves for Early TO failure, FTR, and 30-day mortality are shown in Figure 2.

Observed 30-day mortality proportions and reference mortality estimates across the predefined risk strata are presented descriptively in Supplementary Figure S1. Observed mortality increased across the PULP and PPUMS strata, whereas mortality across the Boey strata showed a less monotonic pattern. Confidence intervals were wide in the highest-risk strata, which contained few patients. Among the 47 patients who developed a qualifying major postoperative complication, 17 were rescued and 30 experienced FTR. Patients who experienced FTR were older and had higher ASA classes, PULP scores, and PPUMSs than rescued patients, whereas Boey scores did not differ between the groups (Table 5). Grade-specific rescue rates were 100.0% (13/13) for Clavien–Dindo grade III, 26.7% (4/15) for grade IVa, and 0.0% (0/19) for grade IVb complications. The distribution of qualifying complication severity differed between the groups, with grade III complications occurring only among rescued patients and grade IVb complications occurring only among patients who experienced FTR.

## 4. Discussion

The principal finding of this study was that the PULP score yielded the numerically highest AUC in each of the three analyses: Early TO failure, FTR, and 30-day mortality. This pattern suggests that PULP may retain prognostic information across the hierarchically related analyses evaluated in this cohort, including major postoperative morbidity and mortality among patients who developed major complications. Although pairwise differences between PULP and PPUMS were not statistically significant for any outcome, this should not be interpreted as evidence of equivalent discriminative performance. The lower discrimination of the Boey score may partly reflect the more limited clinical information captured by its three-component structure compared with PULP and PPUMS. In the present cohort, the three evaluated outcomes were hierarchically related rather than independent. Early TO failure identified the major postoperative morbidity population, FTR represented mortality within this subgroup, and 30-day mortality captured death in the full cohort. Accordingly, FTR provides conditional information among patients with major postoperative complications; however, because these outcomes share common events, consistency of score performance across them should not be interpreted as independent validation across separate outcome domains. In this study, Early TO failure and Clavien–Dindo (CD) grade ≥ III postoperative morbidity overlapped completely at the patient level. This overlap is consistent with the inclusion of CD grade ≥ III postoperative morbidity as a component of the Early TO definition and with the observation that intra-abdominal sepsis, organ failure, unplanned reoperation, and death did not classify any additional patients in this cohort [7]. Thus, Early TO failure did not provide a classification distinct from major postoperative morbidity in the present dataset, and the discriminative performance observed for Early TO failure should be interpreted primarily as reflecting discrimination for major postoperative morbidity rather than performance for a distinct composite postoperative outcome.

Patients with Early TO failure were older and presented with higher ASA classes, higher blood urea nitrogen and creatinine levels, and lower serum albumin levels, suggesting that this outcome may be related to physiological reserve and the degree of systemic derangement at presentation. Similarly, in a recent cohort of patients with perforated peptic ulcer, advanced age, lower serum albumin, and higher creatinine and blood urea nitrogen levels were associated with postoperative morbidity [17]. The absence of between-group differences in perforation size, perforation location, and surgical procedure suggests that the observed separation in this cohort was concentrated more in patient-level physiological characteristics than in anatomical or operative factors. The numerically higher discrimination of PULP for Early TO failure may be related to its broader incorporation of variables reflecting baseline physiological reserve and acute systemic burden.

The PULP and Boey scores have previously been evaluated for overall postoperative morbidity and mortality in perforated peptic ulcer, but they have not been directly compared for FTR [12,13]. Likewise, studies examining high cumulative complication burden and a recent meta-analysis did not evaluate FTR as a distinct outcome [14,15]. To our knowledge, the present study therefore provides the first direct comparative evaluation of these scores for FTR. In our cohort, PULP demonstrated greater discrimination for FTR than the Boey score; however, this finding should be considered exploratory given the limited FTR subgroup and the uncertainty reflected by the corresponding confidence intervals. In unadjusted comparisons, patients with FTR were older and had higher ASA classes, CCI values, and blood urea nitrogen levels than rescued patients. However, complication severity differed markedly between the groups, and these descriptive associations should not be interpreted as independent predictors of FTR. In the major abdominal surgery literature, higher ASA class and Charlson Comorbidity Index have also been associated with FTR [18]. The greater ability of PULP to distinguish this profile compared with Boey may be related to its broader incorporation of age, ASA class, comorbidity domains, and acute physiological derangement. The decline in rescue rates with increasing complication severity further underscores the close relationship between complication severity and rescue status; however, analyses stratified by complication grade should be considered exploratory because of the small subgroup sizes. Given the close relationship between complication severity and rescue status, the FTR findings should be interpreted primarily as discrimination of 30-day mortality among patients with documented major postoperative complications rather than as direct prediction of institutional rescue capability. Because this subgroup was defined by a post-baseline complication, associations between preoperative score components and mortality may differ from those observed in the full cohort. FTR definitions vary across surgical outcomes studies, particularly with respect to the complications included in the denominator and whether postoperative death alone is sufficient for denominator inclusion [19]. In the present cohort, a broader sensitivity definition that included CD grade V deaths irrespective of a separately documented preceding grade III/IV complication identified the same 47 patients and yielded an unchanged FTR rate and identical discrimination estimates, because all 30 deaths were preceded by a documented grade IV complication.

Thirty-day mortality provides an established basis for comparison because it is the outcome for which these scores have been evaluated most extensively. In our cohort, PULP discriminated 30-day mortality with an AUC of 0.851, numerically similar to the AUC of 0.83 reported in its national derivation cohort [5]. Similarly, the AUC of 0.790 for PPUMS was numerically similar to the values of 0.785 and 0.787 reported for in-hospital mortality in the derivation and split-sample internal validation cohorts of the original study [6]. These findings should therefore be interpreted as comparative evidence of discrimination rather than formal external validation, particularly because calibration was not assessed and Early TO failure and FTR were not original score endpoints. The mortality discrimination of the PULP and Boey scores has varied across different populations [20,21]. In Supplementary Figure S1, mortality increased across PULP and PPUMS strata, whereas risk ordering across Boey strata was less consistent. Because the PULP reference values were partly derived from the score distribution of the present cohort, these comparisons should be interpreted descriptively and not as evidence of formal calibration. The Boey score was developed in a younger cohort that included only patients with perforated duodenal ulcers [3]. Differences in age and case mix, together with the small upper-risk strata in the original Boey publication, may have contributed to the differences between published and observed mortality patterns. However, because analyses stratified by age and case mix were not performed, this explanation remains speculative.

PPUMS was developed recently and, to our knowledge, has not been evaluated in an independent cohort beyond the split-sample internal validation reported in the original study [6]. Its performance for Early TO failure and FTR has also not previously been examined; therefore, the present study provides one of the first independent assessments of PPUMS discrimination for outcomes beyond mortality. One possible explanation for the observed numerical ranking is the differing clinical information captured by the three scores. Boey is based on three binary variables, whereas PPUMS incorporates age and selected chronic conditions, and PULP additionally includes indicators of acute physiological derangement. The AUC difference between PPUMS and Boey was statistically significant for FTR but not for Early TO failure after adjustment for multiple comparisons. The absence of direct measures of acute physiological derangement in PPUMS may have contributed to its numerically lower discrimination than PULP. However, particularly in the limited FTR sample, the absence of a significant difference should not be interpreted as evidence that the scores are equivalent.

National trend studies of peptic ulcer disease have shown that the burden of hospitalization and mortality has increasingly shifted toward older patients with greater comorbidity burden [22,23]. However, variable and nonstandardized reporting of patient characteristics and clinical descriptors in studies of perforated peptic ulcer complicates evaluation of the influence of case mix on outcomes [24]. More broadly, outcomes after gastrointestinal perforation are influenced by patient condition at presentation, physiological and septic deterioration, and the timeliness of definitive management [2,25]. In this context, re-examining the performance of previously developed risk scores in contemporary patient populations may be useful. Our findings suggest that PULP, which incorporates age, comorbidity burden, and acute physiological derangement, and PPUMS, which is based on age and selected comorbidities, may retain broader prognostic information about postoperative risk than the three-variable Boey score. However, discrimination alone does not establish calibration or clinical utility. Accordingly, the present findings should be regarded as comparative evidence of discriminative performance rather than as support for direct use of these scores in risk communication or postoperative monitoring. Prospective evaluation incorporating formal calibration and clinical utility analyses is needed to determine their potential clinical role.

### Limitations

The single-center, retrospective design carries risks of selection bias, misclassification, and unmeasured confounding and limits the generalizability of the findings. Retrospective operationalization of selected score components may have introduced misclassification and reduced comparability with the original derivation cohorts. Exclusion of 10 patients because complete calculation of the three prespecified risk scores was not possible may also have introduced selection bias, particularly because several baseline characteristics and 30-day mortality could not be reliably ascertained in the excluded group. The limited size of the FTR subgroup reduced the precision of the AUC estimates and the statistical power to detect differences between the scores. Accordingly, the FTR analyses should be interpreted as exploratory rather than confirmatory. Because eligibility for the primary FTR denominator required a separately documented qualifying complication, retrospective variation in the completeness and timing of postoperative documentation could have affected FTR classification; however, no death was excluded by this requirement in the present cohort. The single-center setting also precluded separation of patient-level risk from center-specific care processes in the FTR analysis. The extended study period may have been associated with temporal changes in perioperative and postoperative management, which could have influenced clinical outcomes, particularly FTR. The complete overlap between Early TO failure and CD grade ≥ III postoperative morbidity in this cohort indicates that the individual components of the composite outcome did not distinguish separate patient groups. Finally, comparisons between observed and reference mortality were limited to descriptive score-stratum summaries. Individual predicted probabilities were unavailable; therefore, calibration intercept and slope could not be estimated, and no formal clinical utility analysis was performed.

## 5. Conclusions

PULP consistently yielded numerically higher AUCs than PPUMS across the three evaluated analyses; however, none of the corresponding PULP–PPUMS differences were statistically significant, and neither superiority nor equivalence can be inferred from these comparisons. In this cohort, Early TO failure completely overlapped with Clavien–Dindo grade ≥ III postoperative morbidity; therefore, its discriminative performance primarily reflected major postoperative morbidity rather than an independent composite outcome. Accordingly, Early TO failure, FTR, and 30-day mortality should be interpreted as hierarchically related analyses rather than as independent postoperative outcome domains. These findings provide comparative evidence of discrimination in this retrospective cohort and should not be interpreted as formal external validation or evidence of clinical utility. Prospective multicenter validation incorporating formal calibration and clinical utility analyses is required before clinical application.

## Figures and Tables

**Figure 1:**
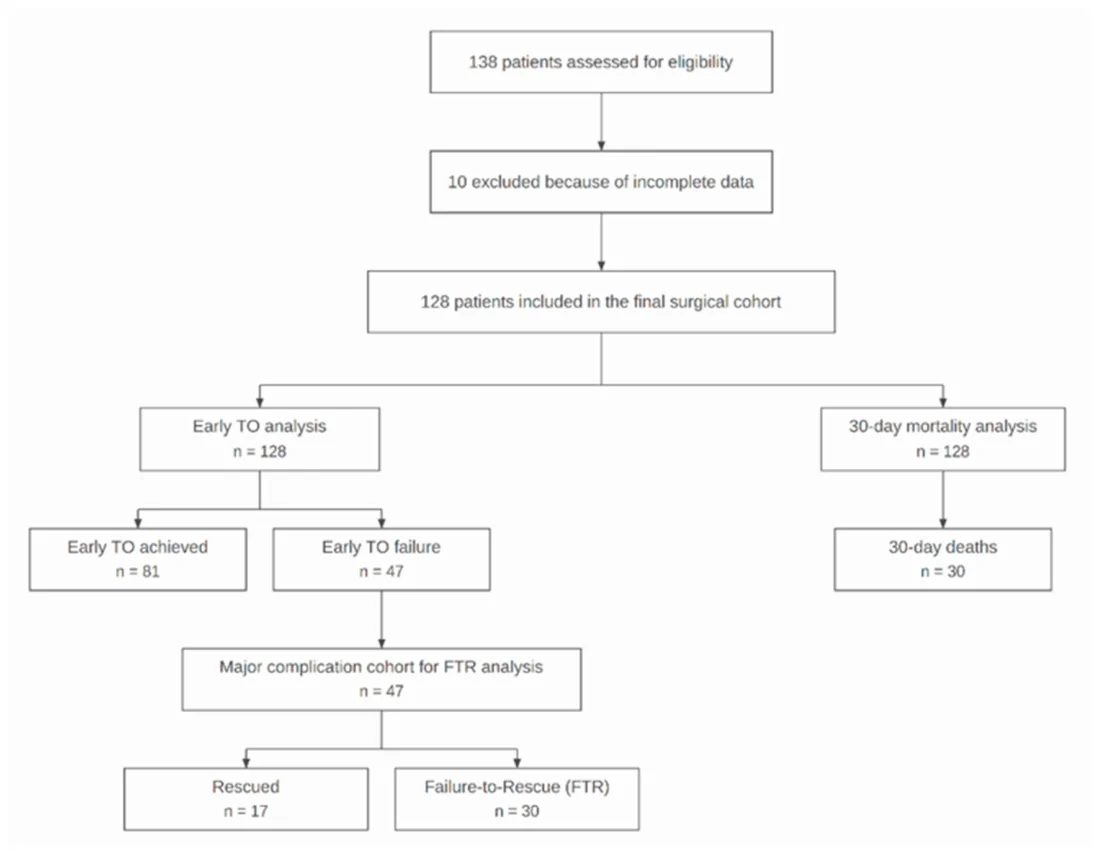
Flowchart of patient selection and outcome-analysis populations. Early TO, Early Textbook Outcome; FTR, Failure-to-Rescue.

**Figure 2:**
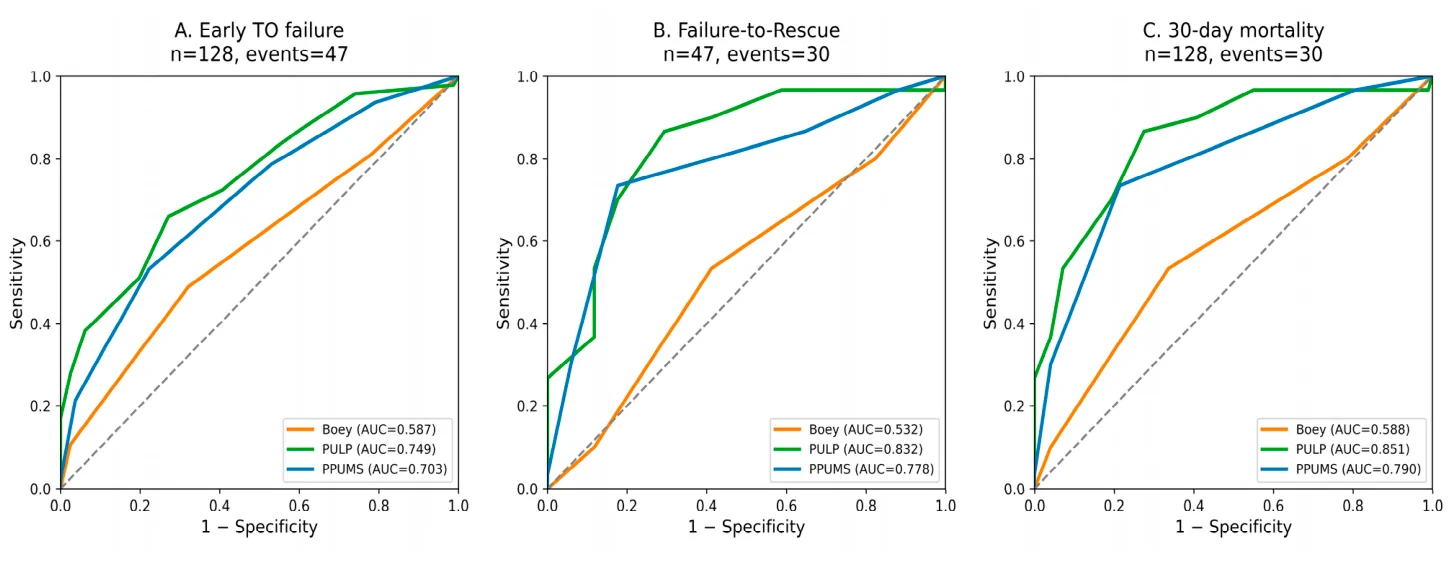
Receiver operating characteristic curves of the Boey, PULP, and PPUMSs for the study outcomes. Panels show discrimination for (**A**) Early TO failure, (**B**) Failure-to-Rescue, and (**C**) 30-day mortality. Early TO failure completely overlapped with CD grade ≥ III postoperative morbidity in this cohort. The diagonal dashed line represents discrimination equivalent to chance. The FTR analysis was restricted to patients with major postoperative complications. Early TO, Early Textbook Outcome; FTR, Failure-to-Rescue; ROC, receiver operating characteristic; AUC, area under the curve; PULP, Peptic Ulcer Perforation score; PPUMS, Perforated Peptic Ulcer Mortality Score.

**Table 1:** Baseline and perioperative characteristics according to Naumann-defined Early Textbook Outcome status.

Variable	Overall (*n* = 128)	Early TO Achieved (*n* = 81)	Early TO Failure (*n* = 47)	*p* Value
Demographic and preoperative characteristics				
Age, years	67 [53–77]	61 [48–70]	76 [63–82.5]	<0.0001
Male sex	88 (68.8)	62 (76.5)	26 (55.3)	0.013
BMI, kg/m^2^	23.2 [20.8–26.0]	23.1 [20.4–25.2]	24.0 [21.2–26.8]	0.140
ASA physical status class	2 [2–3]	2 [2–3]	3 [2–3]	0.0004
Diabetes mellitus	33 (25.8)	19 (23.5)	14 (29.8)	0.430
Congestive heart failure	14 (10.9)	5 (6.2)	9 (19.1)	0.023
COPD	14 (10.9)	7 (8.6)	7 (14.9)	0.275
Chronic kidney disease	7 (5.5)	3 (3.7)	4 (8.5)	0.260
Symptom duration, h	20.5 [10.0–36.0]	18.0 [10.0–30.0]	30.0 [11.0–48.0]	0.288
BUN, mg/dL	23.7 [16.9–35.5]	20.5 [15.8–27.9]	33.2 [25.0–52.3]	<0.0001
Creatinine, mg/dL	1.04 [0.79–1.62]	0.92 [0.74–1.18]	1.49 [1.11–2.28]	<0.0001
Albumin, g/L	30.0 [25.2–34.5]	31.2 [27.0–37.0]	27.0 [22.0–30.8]	0.001
WBC, ×10^3^/µL	10.5 [6.7–14.6]	11.1 [7.3–14.9]	9.3 [5.1–13.0]	0.102
Perforation and operative characteristics				
Perforation size, mm	10.0 [10.0–15.0]	10.0 [10.0–15.0]	10.0 [10.0–17.5]	0.720
Perforation site				0.529
Gastric	94 (73.4)	61 (75.3)	33 (70.2)	
Duodenal	34 (26.6)	20 (24.7)	14 (29.8)	
Open surgical approach	128 (100.0)	81 (100.0)	47 (100.0)	—
Procedure type				0.065
Modified Graham patch repair	119 (93.0)	72 (88.9)	47 (100.0)	
Classic Graham patch repair	4 (3.1)	4 (4.9)	0 (0.0)	
Resection	5 (3.9)	5 (6.2)	0 (0.0)	

Data are presented as median [interquartile range] or *n* (%). Percentages were calculated within columns. Continuous variables were compared using the Mann–Whitney U test. Categorical variables were compared using Pearson’s chi-square test, Fisher’s exact test, or the Fisher–Freeman–Halton exact test, as appropriate. The *p* values for perforation site and procedure type represent comparisons of the overall category distributions. Perforation diameter was converted from centimeters to millimeters. No inferential comparison was performed for surgical approach because all procedures were performed using an open approach. Detailed score-component definitions and prevalences for the Boey, PULP, and PPUMSs are provided in Supplementary Table S2. Early TO, Early Textbook Outcome; BMI, body mass index; ASA, American Society of Anesthesiologists; COPD, chronic obstructive pulmonary disease; BUN, blood urea nitrogen; WBC, white blood cell count.

**Table 2:** Postoperative outcomes, components of Early Textbook Outcome failure, and outcome-analysis populations.

Outcome	Full Cohort or Relevant Population, *n*/*N* (%)	Among Early TO Failures, *n*/*N* (%)
Early TO achieved	81/128 (63.3)	—
Early TO failure	47/128 (36.7)	47/47 (100.0)
Components of Early TO failure †		
CD grade ≥ III postoperative morbidity	47/128 (36.7)	47/47 (100.0)
Intra-abdominal sepsis	29/128 (22.7)	29/47 (61.7)
Organ failure	32/128 (25.0)	32/47 (68.1)
Unplanned reoperation	10/128 (7.8)	10/47 (21.3)
30-day death	30/128 (23.4)	30/47 (63.8)
Additional Early TO failures attributable solely to non-CD ≥ III components	0/128 (0.0)	0/47 (0.0)
Rescued after a major postoperative complication	17/47 (36.2)	—
Failure-to-Rescue	30/47 (63.8)	—
Length of hospital stay, days	9 [6–12]	—

Data are presented as *n*/*N* (%) or median [interquartile range], as appropriate. † Individual Early TO components were not mutually exclusive; patients could meet more than one component. Early TO failure was defined by any of the following: CD grade ≥ III postoperative morbidity, intra-abdominal sepsis, organ failure, unplanned reoperation, or death within 30 days. All 47 patients with Early TO failure also had CD grade ≥ III morbidity, and no additional failures were identified solely by another component. FTR was evaluated among patients with a separately documented CD grade III or IV postoperative complication preceding death or discharge; CD grade V alone did not establish denominator eligibility. No deaths were excluded by this definition because all 30 deaths were preceded by a documented CD grade IV complication. CD, Clavien–Dindo; Early TO, Early Textbook Outcome; FTR, Failure-to-Rescue; IQR, interquartile range.

**Table 3:** Distribution of Boey, PULP, and PPUMSs according to clinical outcomes.

Outcome	Group	Boey Score	PULP Score	PPUMSs
Early TO	Achieved (*n* = 81)	1 [1–2]	4 [1–6]	2 [1–2]
	Failure (*n* = 47)	1 [1–2]	7 [4–9]	3 [2–3]
	*p* value	0.083	<0.0001	<0.0001
Failure-to-Rescue	Rescued (*n* = 17)	1 [1–2]	4 [2–6]	2 [1–2]
	FTR (*n* = 30)	2 [1–2]	8 [6–9.8]	3 [2.3–4]
	*p* value	0.709	0.0002	0.001
30-day mortality	Survived (*n* = 98)	1 [1–2]	4 [2–6]	2 [1–2]
	Died (*n* = 30)	2 [1–2]	8 [6–9.8]	3 [2.3–4]
	*p* value	0.124	<0.0001	<0.0001

Data are presented as median [interquartile range]. Comparisons were performed using the two-sided Mann–Whitney U test. The FTR comparison was restricted to the 47 patients who developed a major postoperative complication. Early TO, Early Textbook Outcome; FTR, Failure-to-Rescue; PULP, Peptic Ulcer Perforation score; PPUMS, Perforated Peptic Ulcer Mortality Score.

**Table 4:** Discriminative performance of the risk scores and pairwise comparisons of the areas under the receiver operating characteristic curves.

Outcome	Boey, AUC (95% CI)	PULP, AUC (95% CI)	PPUMS, AUC (95% CI)	PULP vs. Boey, ΔAUC (Unadjusted 95% CI); Holm-Adjusted *p*	PPUMS vs. Boey, ΔAUC (Unadjusted 95% CI); Holm-Adjusted *p*	PULP vs. PPUMS, ΔAUC (Unadjusted 95% CI); Holm-Adjusted *p*
Early Textbook Outcome failure/CD grade ≥ III morbidity, 47/128	0.587 (0.487–0.687)	0.749 (0.660–0.838)	0.703 (0.611–0.795)	0.162 (0.060–0.265); 0.006	0.116 (0.012–0.221); 0.057	0.046 (−0.014–0.106); 0.135
Failure-to-Rescue, 30/47	0.532 (0.365–0.700)	0.832 (0.706–0.959)	0.778 (0.642–0.914)	0.300 (0.136–0.464); 0.001	0.246 (0.088–0.404); 0.004	0.054 (−0.063–0.171); 0.366
30-day mortality, 30/128	0.588 (0.469–0.707)	0.851 (0.768–0.934)	0.790 (0.695–0.885)	0.263 (0.155–0.371); <0.0001	0.202 (0.093–0.312); 0.0006	0.061 (−0.007–0.128); 0.079

Values accompanying each outcome indicate the number of events divided by the corresponding analysis population. Pairwise comparisons of correlated AUCs were performed using the paired DeLong test. AUC differences are presented with 95% confidence intervals, and Holm-adjusted *p* values are reported for each comparison. Holm adjustment was applied separately within each outcome across the three pairwise comparisons. Unadjusted *p* values are reported in the Results text. Bold values indicate statistical significance based on Holm-adjusted *p* < 0.05. The Failure-to-Rescue analysis was restricted to the 47 patients with a documented Clavien–Dindo grade III or IV postoperative complication. In this cohort, Early Textbook Outcome failure completely overlapped with Clavien–Dindo grade ≥ III postoperative morbidity at the patient level. The 95% confidence intervals for the AUC differences were unadjusted. AUC, area under the receiver operating characteristic curve; CI, confidence interval; PULP, Peptic Ulcer Perforation score; PPUMS, Perforated Peptic Ulcer Mortality Score.

**Table 5:** Exploratory comparison of patients who were rescued versus those who experienced Failure-to-Rescue.

Variable	Rescued (*n* = 17)	Failure-to-Rescue (*n* = 30)	Rescue Rate Within Grade, *n*/*N* (%)	*p* Value
Preoperative clinical and laboratory characteristics				
Age, years	64 [53–70]	78 [73.3–85]	—	0.0002
ASA physical status class	2 [2–2]	3 [3–3]	—	0.0005
Charlson Comorbidity Index, age-unadjusted	0 [0–0]	2 [0–3]	—	0.002
Albumin, g/L	27.4 [24.0–32.0]	25.9 [20.3–30.0]	—	0.177
BUN, mg/dL	20.9 [19.7–35.4]	36.1 [27.6–56.6]	—	0.012
Creatinine, mg/dL	1.20 [0.93–2.30]	1.77 [1.24–2.23]	—	0.382
Preoperative shock, *n* (%)	2 (11.8)	4 (13.3)	—	1.000
Risk scores				
PULP score	4 [2–6]	8 [6–9.8]	—	0.0002
PPUMS	2 [1–2]	3 [2.3–4]	—	0.001
Boey score	1 [1–2]	2 [1–2]	—	0.709
Major postoperative complication grade, *n* (%)				
Clavien–Dindo grade III	13 (76.5)	0 (0.0)	13/13 (100.0)	—
Clavien–Dindo grade IVa	4 (23.5)	11 (36.7)	4/15 (26.7)	—
Clavien–Dindo grade IVb	0 (0.0)	19 (63.3)	0/19 (0.0)	—

Data are presented as median [interquartile range] or *n* (%), unless otherwise stated. The analysis was restricted to the 47 patients who developed a qualifying major postoperative complication classified as Clavien–Dindo grade III or IV. The Charlson Comorbidity Index was calculated without age adjustment. Continuous and ordinal variables were compared using the two-sided Mann–Whitney U test, and preoperative shock was compared using Fisher’s exact test. Preoperative shock was defined as a systolic blood pressure < 100 mmHg accompanied by a heart rate > 100 beats/min at admission. Between-group comparisons were exploratory and unadjusted and were not adjusted for multiplicity. No multivariable analysis was performed; therefore, these comparisons should not be interpreted as identifying independent predictors of FTR. Grade-specific rescue rates were calculated as the number of rescued patients divided by the total number of patients within each qualifying complication grade and are presented descriptively; no significance test was performed. For patients who experienced Failure-to-Rescue, the qualifying grade refers to a separately documented Clavien–Dindo grade III or IV complication occurring before death; death classified solely as Clavien–Dindo grade V was not sufficient to establish eligibility. ASA, American Society of Anesthesiologists; BUN, blood urea nitrogen; PULP, Peptic Ulcer Perforation score; PPUMS, Perforated Peptic Ulcer Mortality Score; IQR, interquartile range.

## Data Availability

The raw data supporting the conclusions of this article will be made available by the authors on request.

## Supplementary Materials

The following supporting information can be downloaded at https://www.mdpi.com/article/10.3390/medicina62101847/s1: Table S1: Missing-data profile of excluded patients and comparison of available characteristics between included and excluded patients; Table S2: Original score components, operational definitions, data sources, and component prevalence in the study cohort; Figure S1: Descriptive comparison of observed and reference mortality across risk strata of the Boey, PULP, and PPUMS scores.
