# Supplementary material for: Comparative Discrimination of Boey, PULP, and PPUMSs for Textbook Outcome Failure, Failure-to-Rescue, and 30-Day Mortality Following Perforated Peptic Ulcer Surgery
Source: Medicina (Kaunas). 2026 Sep 24;62(10):1847. doi: 10.3390/medicina62101847 (PMC13706765; doi:10.3390/medicina62101847)
Supplement: Supplementary file 1 [file medicina-62-01847-s001.zip › medicina-4522770-supplementary.pdf]

Supplementary Table S1. Missing-data profile of excluded patients and comparison of available characteristics between included and excluded patients

Panel A. Missing variables among the 10 patients excluded because complete risk-score calculation was not possible

Variable unavailable	Missing among excluded patients, n/10 (%)	Risk score(s) affected
Body mass index	10/10 (100.0)	PPUMS
Steroid use	10/10 (100.0)	PULP
Symptom-onset timing information required for score calculation*	9/10 (90.0)	Boey, PULP
Admission systolic blood pressure	8/10 (80.0)	Boey, PULP
Admission heart rate	8/10 (80.0)	PULP
Serum creatinine	8/10 (80.0)	PULP
ASA physical status	6/10 (60.0)	PULP
Diabetes mellitus status	6/10 (60.0)	Boey
Coronary artery disease/previous myocardial infarction status	6/10 (60.0)	Boey
Congestive heart failure status	6/10 (60.0)	Boey, PPUMS
Chronic obstructive pulmonary disease status	6/10 (60.0)	Boey
Chronic kidney disease status	6/10 (60.0)	Boey, PPUMS
Cirrhosis status	6/10 (60.0)	PULP
Severe liver disease status	6/10 (60.0)	PPUMS
Active malignancy/AIDS status	6/10 (60.0)	PULP
History of malignancy	6/10 (60.0)	PPUMS
At least one missing variable precluding complete calculation of all three prespecified scores	10/10 (100.0)	Boey/PULP/PPUMS

* *Symptom-onset timing information refers to the timing variables required to determine the perforation-duration components of the Boey and PULP scores.*

Panel B. Comparison of available characteristics between included and excluded patients

Characteristic	Included patients (n = 128)	Excluded patients (n = 10)	Available n in excluded group	p value
Age, years	67 [53–77]	56.0 [54.0–62.5]	10/10	0.115
Male sex	88 (68.8)	5 (50.0)	10/10	0.294
ASA physical status	2 [2–3]	2 [2–2.25]	4/10	—
Gastric perforation	94 (73.4)	3/4 (75.0)	4/10	—
Diabetes mellitus	33 (25.8)	4/4 (100.0)	4/10	—
Congestive heart failure	14 (10.9)	1/4 (25.0)	4/10	—
Chronic obstructive pulmonary disease	14 (10.9)	0/4 (0.0)	4/10	—
Chronic kidney disease	7 (5.5)	0/4 (0.0)	4/10	—
In-hospital mortality	30 (23.4)	0 (0.0)	10/10	0.118

Data are presented as median [interquartile range] or n (%), as appropriate. Patients could have more than one missing variable; therefore, counts in Panel A are not mutually exclusive and may sum to more than 10. Patients were excluded when incomplete retrospective records precluded reliable calculation of at least one of the three prespecified risk scores required for the comparative analysis. No imputation was performed. Comparisons in Panel B were restricted to characteristics available in the excluded patients, and the number of available observations is reported for each variable. Formal hypothesis testing was limited to characteristics available for all 10 excluded patients; variables available in only four patients are presented descriptively. Continuous variables were compared using the two-sided Mann–Whitney U test and categorical variables using Fisher's exact test. Thirty-day mortality could not be reliably ascertained for the excluded patients; therefore, the mortality comparison was limited to documented in-hospital mortality. ASA, American Society of Anesthesiologists; COPD, chronic obstructive pulmonary disease; PULP, Peptic Ulcer Perforation score; PPUMS, Perforated Peptic Ulcer Mortality Score.

Supplementary Table S2. Original score components, operational definitions, data sources, and component prevalence in the study cohort

Score	Original component	Operational definition in the present study	Data source	Prevalence, n/N (%)
Boey	Major medical illness	Presence of ≥1 of the following: coronary artery disease or previous myocardial infarction, congestive heart failure, chronic obstructive pulmonary disease, pre-existing chronic kidney disease, diabetes mellitus, or documented hepatic pre-coma/encephalopathy. Liver cirrhosis without hepatic pre-coma or encephalopathy did not qualify.	Electronic medical records and archived patient files	56/128 (43.8%)

Boey	Preoperative shock	Systolic blood pressure <100 mmHg at hospital admission	Admission vital-sign records	35/128 (27.3%)
Boey	Longstanding perforation	>24 h from onset of symptoms consistent with acute perforation to surgery	Emergency admission and operative records	66/128 (51.6%)
PULP	Age >65 years	Age >65 years	Demographic data in electronic medical records	67/128 (52.3%)
PULP	Active malignant disease or AIDS	Documented active malignant disease or acquired immunodeficiency syndrome	Electronic medical records and archived patient files	17/128 (13.3%)
PULP	Liver cirrhosis	Documented liver cirrhosis	Electronic medical records and archived patient files	2/128 (1.6%)
PULP	Concomitant steroid use	Documented concomitant steroid use at presentation	Medication history in electronic medical records and archived patient files	10/128 (7.8%)
PULP	Shock on admission	Systolic blood pressure <100 mmHg with concurrent heart rate >100 beats/min at hospital admission	Admission vital-sign records	18/128 (14.1%)
PULP	Time from perforation to admission >24 h	>24 h from onset of symptoms consistent with acute perforation to hospital admission	Emergency admission records	62/128 (48.4%)
PULP	Serum creatinine >130 μmol/L	First available preoperative serum creatinine >130 μmol/L (approximately >1.47 mg/dL)	First available preoperative laboratory results	37/128 (28.9%)
PULP	ASA physical status	ASA classes I, II, III, IV, and V were assigned 0, 1, 3, 5, and 7 points, respectively	Preoperative anesthesia and clinical records	ASA I: 9/128 (7.0%); ASA II: 69/128 (53.9%); ASA III: 45/128 (35.2%); ASA IV: 5/128 (3.9%); ASA V: 0/128 (0.0%)
PPUMS	Age category	Mutually exclusive age categories were operationalized as <45, 45–64, 65–80, and >80 years	Demographic data in electronic medical records	<45: 20/128 (15.6%); 45–64: 39/128 (30.5%); 65–80: 49/128 (38.3%); >80: 20/128 (15.6%)

PPUMS	Congestive heart failure	Documented pre-existing congestive heart failure	Electronic medical records and archived patient files	14/128 (10.9%)
PPUMS	Severe liver disease	Cirrhosis with at least one of the following: intractable ascites, hepatic encephalopathy, or esophageal variceal bleeding	Electronic medical records and archived patient files	0/128 (0.0%)
PPUMS	Renal disease	Pre-existing chronic kidney disease was used as the clinical operationalization of the original comorbidity-based renal-disease component. Isolated elevation in serum creatinine at admission did not qualify.	Electronic medical records and archived patient files	7/128 (5.5%)
PPUMS	History of malignancy	Documented history of malignancy	Electronic medical records and archived patient files	16/128 (12.5%)
PPUMS	Obesity	Body mass index ≥30 kg/m² was used as the clinical operationalization of the original diagnosis-code-based obesity component	Recorded height and weight used to calculate body mass index	11/128 (8.6%)

Data are presented as n/N (%) unless otherwise indicated. Prevalence was calculated in the full analytic cohort (N=128). For multilevel components, category distributions are reported. The original Boey score comprises major medical illness, preoperative shock, and longstanding perforation (>24 h); in the present retrospective dataset, these components were operationalized using the clinical variables specified above. The two patients with liver cirrhosis fulfilled the PULP cirrhosis component; however, neither met the PPUMS definition of severe liver disease, which required cirrhosis with intractable ascites, hepatic encephalopathy, or esophageal variceal bleeding. Accordingly, the prevalence of the PPUMS severe-liver-disease component was 0/128. For PPUMS, renal disease and obesity were originally identified from comorbidity/diagnostic-code data; in the present clinical dataset, these components were operationalized as pre-existing chronic kidney disease and body mass index ≥30 kg/m², respectively. AIDS, acquired immunodeficiency syndrome; ASA, American Society of Anesthesiologists; BMI, body mass index; PULP, Peptic Ulcer Perforation score; PPUMS, Perforated Peptic Ulcer Mortality Score.

Supplementary Figure S1: Descriptive comparison of observed and reference mortality across risk strata of the Boey, PULP, and PPUMS scores.

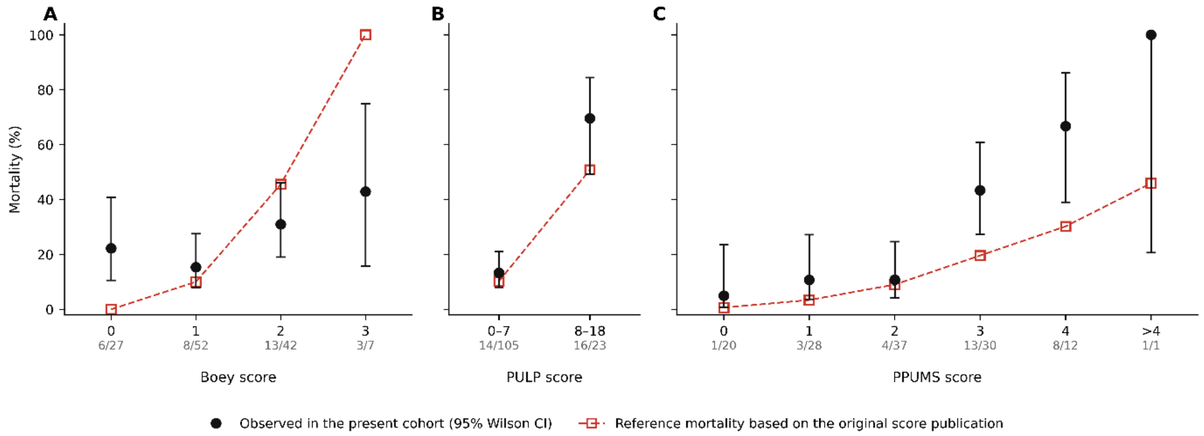

Figure S1. Descriptive comparison of observed and reference mortality across risk strata of the Boey, PULP, and PPUMS scores. Panels show mortality according to (A) Boey score, (B) PULP risk category, and (C) PPUMS score. Black circles indicate observed 30-day mortality in the present cohort, with 95% Wilson confidence intervals. Red open squares connected by dashed lines indicate reference mortality based on the original score publications [3,5,6]. For PULP, category-level reference mortality was reconstructed by weighting published score-specific mortality estimates according to the distribution of PULP scores within each category in the present cohort; these reference values therefore partly reflect the score distribution of the current cohort.Deaths/total patients are shown below each stratum. In the present cohort, all deaths occurred during the index hospitalization. Higher-risk strata contained few patients, resulting in imprecise observed mortality estimates; these descriptive comparisons should therefore be interpreted cautiously and do not constitute a formal calibration assessment.
